# The Microbiota and Abundance of the Class 1 Integron-Integrase Gene in Tropical Sewage Treatment Plant Influent and Activated Sludge

**DOI:** 10.1371/journal.pone.0131532

**Published:** 2015-06-26

**Authors:** Magna C. Paiva, Marcelo P. Ávila, Mariana P. Reis, Patrícia S. Costa, Regina M. D. Nardi, Andréa M. A. Nascimento

**Affiliations:** 1 Departamento de Microbiologia, Instituto de Ciências Biológicas, Universidade Federal de Minas Gerais, Belo Horizonte, Minas Gerais, Brazil; 2 Campus Dona Lindu, Universidade Federal de São João del Rei, Divinópolis, Minas Gerais, Brazil; 3 Departamento de Biologia Geral, Instituto de Ciências Biológicas, Universidade Federal de Minas Gerais, Belo Horizonte, Minas Gerais, Brazil; Cairo University, EGYPT

## Abstract

Bacteria are assumed to efficiently remove organic pollutants from sewage in sewage treatment plants, where antibiotic-resistance genes can move between species via mobile genetic elements known as integrons. Nevertheless, few studies have addressed bacterial diversity and class 1 integron abundance in tropical sewage. Here, we describe the extant microbiota, using V6 tag sequencing, and quantify the class 1 integron-integrase gene (*intI1*) in raw sewage (RS) and activated sludge (AS). The analysis of 1,174,486 quality-filtered reads obtained from RS and AS samples revealed complex and distinct bacterial diversity in these samples. The RS sample, with 3,074 operational taxonomic units, exhibited the highest alpha-diversity indices. Among the 25 phyla, Proteobacteria, Bacteroidetes and Firmicutes represented 85% (AS) and 92% (RS) of all reads. Increased relative abundance of Micrococcales, Myxococcales, and Sphingobacteriales and reduced pathogen abundance were noted in AS. At the genus level, differences were observed for the dominant genera *Simplicispira* and *Diaphorobacter* (AS) as well as for *Enhydrobacter* (RS). The activated sludge process decreased (55%) the amount of bacteria harboring the *intI1* gene in the RS sample. Altogether, our results emphasize the importance of biological treatment for diminishing pathogenic bacteria and those bearing the *intI1* gene that arrive at a sewage treatment plant.

## Introduction

Although water is fundamental to life on earth and is considered a renewable and infinite resource, it is still limited. Wastage, environmental imbalances and water pollution due to domestic and industrial sewage threaten the availability of this natural resource, which is in high demand. The contemporary world has contributed to environmental pollution by releasing large amounts of sewage into waterways, leading to human exposure and the contamination of several environments. In Brazil, approximately 8 billion liters of non-treated sewage is released into rivers each day from one hundred of the largest Brazilian cities [1]. This represents a environmental challenge because the discharge of untreated or improperly treated waste into waterways can introduce potentially pathogenic microorganisms to humans and change the indigenous microbial community, with ecological and public health implications [2]. Notably, the aquatic environment harbors different antibiotic resistance-associated mobile genes that are scattered among environmental bacteria [3]. As outlined in a review by Gillings *et al*. [4], class 1 integron-integrase is consistently linked to genes that confer resistance to antibiotics, disinfectants and heavy metals, is found in pathogenic and commensal bacterial species of humans and animals and is able to move between species. Wastewater discharge, manure disposal and aquaculture are the main sources of antibiotics in aquatic environments [5]. Previous studies have agreed that the microbiota of wild animals can acquire antibiotic resistance genes by consuming waste or drinking contaminated water [6, 7].

Efforts to treat sewage are occurring worldwide. Among these processes, aerobic biological treatment by activated sludge (AS) has been successfully applied and widely accepted for treating domestic sewage. This system represents an environmental protection and offers a low-cost and effective way to treat sewage [8], with the removal of over 90% of organic material [9, 10]. However, the efficacy of this treatment depends on a series of metabolic interactions among diverse microorganisms, mostly bacteria, that play a key role to remove organic and inorganic pollutants.

Many studies based on 16S rRNA gene analysis have described microbial groups found in anaerobic reactors and AS [11–13], but less is known regarding microbiota from raw sewage (RS) and the abundance of the class 1 integron-integrase gene (*intI1*) in this environment. Knowledge on this microbiota is important because it may identify bacteria that can harm human health. Additionally, the distribution of class 1 integrons from metagenomic samples, in contrast to culturable bacteria, remains under-explored. Thus, evaluating the abundance of class 1 integrons, which are often associated with multiresistant clinical pathogens, is important [14, 7]. To gain insight into this knowledge gap, we investigated the bacterial diversity of RS and AS of a full-scale activated sludge system using high-throughput sequencing. Moreover, we also investigated the abundance of the 16S rRNA and *intI1* genes in these environments using real-time PCR.

## Materials and Methods

### Ethics statement

For sampling in the Arrudas wastewater treatment plant, no specific permit was required for the described study area and we confirm that it did not involve endangered or protected species.

### Study area and sampling

The Arrudas wastewater treatment plant, Belo Horizonte, Brazil (19°53’42”S and 43°52’42”W) occupies an area of 63.84 ha, and treats the domestic sewage (2.25 m^3^/s design flow) generated by 1 million inhabitants using a conventional activated sludge process. Samples from sewage and wastewater treatment station represent a combination of inputs from human faecal microbes and enrichment of specific microbes from the environment to form a unique and stable population structure [15].

RS (10 L) and AS (2 L) samples were placed in sterilized bottles on 7 May 2013 and transported on ice to the laboratory within 2 h. The AS sample corresponded to a mixed liquid containing flocs and suspended bacteria from the aerobic zone of the aeration tank. The samples were then centrifuged at 14,000x g for 10 min, and the supernatants were discharged and the final pellets were stored at -20°C until further processing.

### DNA extraction and sequencing

Total DNA was extracted from 10 g of wet pellets stored at -20°C after thawing at room temperature, using the PowerSoil DNA Isolation Kit (Mobio Laboratories Inc., Carlsbad, CA, USA) according to the manufacturer's instructions. The quantity and quality of the total DNA were determined using a NanoDrop spectrophotometer (NanoDrop Technologies).

Partial amplification of the 16S rRNA gene was achieved using the primer set 985F (5’-CAACGCGAAGAACCTTAC C-3’) and 1046R (5’-CGACAGCCATGCANCACCT-3’) [16], which corresponded to the V6 hypervariable region. Gene amplification and sequencing were performed at the Beijing Genomics Institute (BGI), using the 100 bp paired-end strategy on the Illumina HiSeq 2000 platform.

### Data analysis and taxonomic assignment

In this study, reads assembly, trimming and screening were carried out using Mothur v.1.32.0 [17]. Sequences with low quality (≤20) or ambiguous bases and with more than eight homopolymers and a read length outside the range of 56–63 bp were discarded. Reads approved using these quality criteria were aligned and classified against a V6 region trimmed alignment that was based on the Silva v.119 16S rRNA database [18]. Chimeric reads were identified and excluded using the Uchime method [19], and mitochondrial and chloroplast reads and reads that did not match any reference sequence from the bacterial 16S rRNA database were discarded. Subsequently, the reads were grouped into operational taxonomic units (OTUs) by considering a genetic similarity of 95% using the average neighbor method. The samples were normalized to the lowest number of reads using the command rarefy_even_depth with the Phyloseq [20] package of the R software [21] to determine the alpha diversity indices. The nucleotide sequences were submitted to Sequence Read Archive (http://www.ncbi.nlm.nih.gov/sra/) with the accession numbers of SRR 1801880 to SRR 1801935.

### Quantitative real-time PCR (qPCR)

For each RS and AS sample, we estimated the abundance of the bacterial 16S rRNA and *intI1* genes using an ABI PRISM 7900HT sequence detection system (Applied Biosystems, Foster City, CA). The primer sets 338F (5’-TACGGGAGGCAGCAG-3’) [22] and 518R (5’-ATTACCGCGGCTGCTGG-3’) [23] and qINT-3 (5’-TGCCGTGATCGAAATCCAGATCCT-3’) and qINT-4 (5’-TTTCTGGAAGGCGAGCATCGTTTG-3’) [24] were used to amplify the 16S rRNA and *intI1* genes, respectively. The amplification reaction conditions were described by Reis *et al*. [25] and Rosewarne *et al*. [24], respectively. Standard curves were generated using seven dilutions, in triplicate, of the 16S rRNA and *intI* amplicons from the *Escherichia coli* ATCC 25922 (3.82 x 10^11^ copies/μL) and *E*. *coli* BH100 strains (3.20 x 10^12^ copies/μL). To determine the number of the 16S rRNA and *intl1* genes copies used as templates for the standard curves, the following online calculator was accessed: http://www.uri.edu/research/gsc/resources/cndna.html [26].

Each sample was run in triplicate, and a negative control was included for each analysis. The standard curves for the primer sets generated slopes of -3.19 and -3.27, respectively, and the *R* ^*2*^ values were greater than 0.97 for both curves (S1 Fig). Bacterial qPCR exhibited *C*t values of 10.7 and 12.50 for RS and AS, respectively. In contrast, the *C*t values obtained for *intI1* were 16.81 and 19.68 for RS and AS, respectively (S2 Fig). The proportion of bacterial cells harboring the *intI1* gene in each sample was estimated as previously described by Hardwick *et al*. [27].

## Results and Discussion

### Overview of the datasets and alpha diversity

Illumina-based V6 tag sequencing yielded 1,885,944 raw reads (943,000 in RS and 942,944 in AS, 339.47 Mb). The resultant clean reads (598,119 in RS and 587,243 in AS, with an average length of 61 bp) were used for downstream analysis. There were a total of 3,074 (RS) and 1,952 (AS) OTUs (S1 Table).

**Fig 1:**
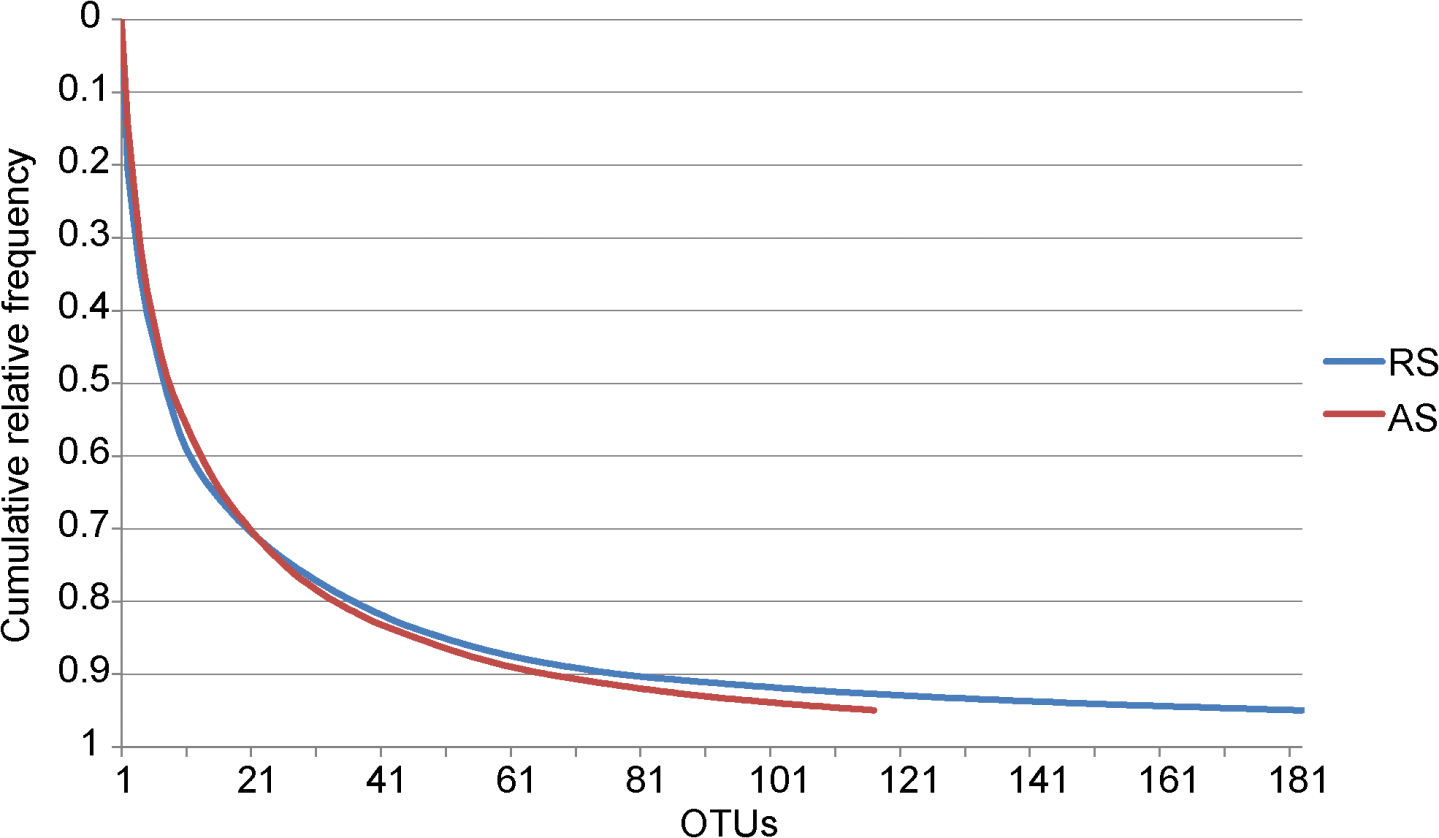
Cumulative relative frequency of the OTUs of raw sewage (RS) and activated sludge (AS).

To estimate the relative diversity captured in each sample, the normalized libraries (with the lowest number of reads, 587,243) were used to calculate the cumulative relative frequency curve, OTU richness, ChaoI and ACE richness estimators, as well as the Shannon and Simpson diversity indices. Good’s coverage values were similar in both samples (RS, 99.85%; AS, 99.9%). As illustrated in Fig 1, the bacterial communities from RS and AS were dominated by a few OTUs. The long tails of the taxon rank distribution curves indicated that the diversity in these environments mostly arose from rare taxa. Although both communities exhibited a high number of rare OTUs, the read distribution of dominant OTUs in each sample was relatively equitable, as shown by the high Simpson index values (RS, 0.93; AS, 0.95). Altogether, Good’s coverage and the cumulative relative frequency curve suggested that most of the diversity was captured. The Shannon index (RS, 3.96; AS, 3.9) revealed considerable bacterial diversity in the samples. Moreover, the Chao1 (RS, 3677.6; AS, 2346.2) and ACE (RS, 3843.4; AS, 2441.0) values also predicted higher bacterial diversity in the RS sample (S2 Table).

### Taxonomic composition

**Fig 2:**
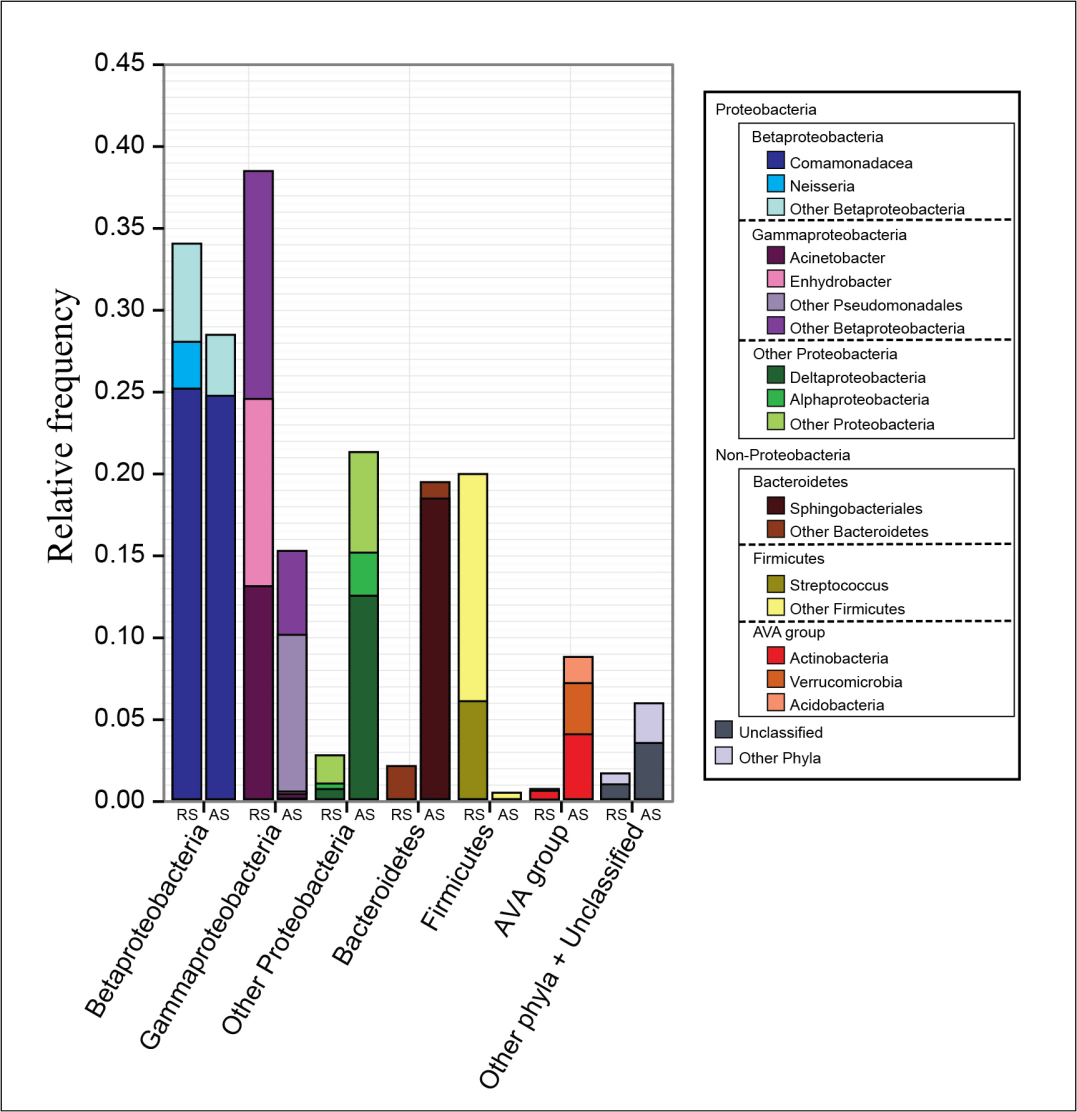
Distribution of the community members in raw sewage (RS) and activated sludge (AS). AVA group: Actinobacteria, Verrucomicrobia, Acidobacteria. Other phyla: BD1-5, Candidate_division_OD1, Candidate_division_OP11, Candidate_division_OP8, Candidate_division_TM7, Candidate_division_WS3, Candidate_division_WS6, Chlamydiae, Chlorobi, Cyanobacteria, Elusimicrobia, Fusobacteria, Gemmatimonadetes, Lentisphaerae, Nitrospirae, SHA-109, Spirochaetae, Tenericutes.

The taxonomic diversity profile by V6 tag sequencing is shown in Fig 2 and S3 Table. The 1,174,486 reads were affiliated with 25 bacterial phyla. Additionally, 1,025 OTUs were considered to be unclassified at the phylum level and thus might represent new bacterial taxa. Importantly, three phyla (Proteobacteria, Bacteroidetes and Firmicutes) accounted for 97% and 85% all of the reads in the RS and AS samples, respectively. Although the RS and AS samples revealed similar phylum-level representation, distinct distributions were observed. Previous studies of microbial sewage and activated sludge communities also revealed a predominance of these phyla [8, 11, 15]; however, they showed a lower proportion of Proteobacteria (36%-65%) than that observed in our study. In contrast to our observations, Firmicutes was previously found in low abundance in the RS [8] and in high abundance in the AS [28]. Moreover, the abundance of Bacteroidetes ranged from 2.7% to 15.6% in activated sludge samples from 14 sewage treatment plants [11]. This difference between our data and those of previous studies can be due to differences in sewage composition because of climatic, geographical and population conditions [12, 29] as well as because of organic loading, pH, temperature, dissolved oxygen and sludge retention time applied in the aeration tank [12, 30].

The compositions of the bacterial communities were distinct and represented by broad intra-phylum diversity. A total of 147 (127 from RS and 113 from AS) families comprising 307 (282 from RS and 207 from AS) genera were identified in both environments. Proteobacteria, especially Beta- and Gammaproteobacteria, were the dominant community members (Fig 2). Comamonadaceae (12.6% from RS and 12.4% from AS) and Pseudomonadales (12.4% from RS and 4.9% from AS) were the predominant taxa in both samples, covering 42.2% of all reads. Betaproteobacteria constitute a major fraction of the microbial community in the activated sludge of domestic sewage treatment plants [31–33], suggesting that their physiological characteristics are functionally relevant for this ecosystem. Moreover, members of the Comamonadaceae family, which play an important role in nutrient removal, were among the most dominant taxa in wastewater treatment systems [15, 32]. Here, two dominant genera, *Simplicispira* and *Diaphorobacter*, were found in AS; these genera have been recovered from activated sludge systems and comprise denitrifying bacteria [34].

Within the Gammaproteobacteria class changes in the bacterial community composition between the samples were observed. Interestingly, considerable enrichment of an OTU (Otu00002) classified only at the Pseudomonadales order level was found in AS (Table 1). Thus, this taxon likely has an ecologically significant role in reducing environmental pollution in wastewater treatment plants. Moreover, the *Enhydrobacter* genus was overrepresented in the RS sample, but information on *Enhydrobacter* is scarce and comprises that of only one species (*Enhydrobacter aerosaccus*) [35] found in primary influent sewage samples [36].

**Table 1:** The top 21 OTUs classified in raw sewage (RS) and activated sludge (AS); (-) unclassified.

OTU	Phylum	Class	Order	Family	Genus	Number of reads RS	Number of reads AS
Otu00029	Acidobacteria	Holophagae	Holophagales	Holophagaceae	*Geothrix*	0	8025
Otu00024	Actinobacteria	Actinobacteria	Micrococcales	Intrasporangiaceae		37	10356
Otu00006	Bacteroidetes	Sphingobacteriia	Sphingobacteriales	Chitinophagaceae		0	35350
Otu00013				NS11-12marine group		0	19713
Otu00021				Chitinophagaceae		0	11502
Otu00022		Flavobacteriia	Flavobacteriales	Flavobacteriaceae	*Cloacibacterium*	8849	1912
Otu00012	Firmicutes	Bacilli	Lactobacillales	Streptococcaceae	*Streptococcus*	21360	315
Otu00017						12806	106
Otu00001	Proteobacteria	Betaproteobacteria	Burkholderiales	Comamonadaceae		121196	84732
Otu00002		Gammaproteobacteria	Pseudomonadales			8	53118
Otu00003				Moraxellaceae	*Enhydrobacter*	45770	727
Otu00004		Deltaproteobacteria				0	46424
Otu00005		Gammaproteobacteria	Pseudomonadales	Moraxellaceae	*Acinetobacter*	40636	1607
Otu00007						30397	2027
Otu00008		Betaproteobacteria	Burkholderiales	Comamonadaceae		2720	24761
Otu00009						5	23976
Otu00010		Gammaproteobacteria				20329	2325
Otu00011			Pseudomonadales	Moraxellaceae	*Enhydrobacter*	21462	257
Otu00014		Betaproteobacteria	Neisseriales	Neisseriaceae	*Neisseria*	16592	398
Otu00015		Gammaproteobacteria				16384	300
Otu00016		Deltaproteobacteria	Myxococcales	Polyangiaceae	*Sorangium*	0	14549

The Alpha- and Deltaproteobacteria classes were found at very low abundances in RS (0.4% and 0. 8%, respectively), whereas they were enriched in AS (2.9% and 12.7%, respectively). Among the 10 most abundant alpha- and deltaproteobacterial OTUs, the Caulobacteraceae family (Alphaproteobacteria) and the Myxococcales (Deltaproteobacteria) order were identified. The presence of *Caulobacter* species in AS is intriguing because these organisms are typically found in water and are considered to be oligotrophic (i.e., adapted to conditions with low nutrient availability) [37, 38]. Myxobacteria are known to be micropredators [39, 40] that are highly effective at degrading organic matter and are found in various habitats but mostly on decaying organic material [37].

The abundance of Firmicutes in AS was significantly lower (0.6%) than in RS (20%), whereas the abundance of Bacteroidetes was greater in AS (19%) than in RS (2%). Previous studies have suggested that Firmicutes species do not thrive in intense aeration conditions, e.g. in activated sludge [8, 41]. The majority of Bacteroidetes were represented by the Flavobacteriales (RS) and Sphingobacteriales (AS) orders, the latter of which are widely abundant in AS plants and metabolize macromolecules such as polysaccharides and proteins [42].

Actinobacteria, Acidobacteria and Verrucomicrobia were the minor phyla and were found mostly in the AS sample. Notably, however, three of the 30 top OTUs of all of the phyla were assigned to the Intrasporangiaceae family (Actinobacteria) and the *Geothrix* (Acidobacteria) and *Prosthecobacter* (Verrucomicrobia) genera. Interestingly, Intrasporangiaceae and *Geothrix* harbor species that accumulate polyphosphates [43] and that participate in the biogeochemical recycling of metals [44], respectively. The genus *Prosthecobacter* has been recovered from activated sludge and can use algal metabolites as carbon and nutrient sources [45, 46].

Interestingly, we found a few pathogen-related OTUs. Among the top 21 OTUs classified at the genus level, *Neisseria*, *Acinetobacter* and *Streptococcus*, which are known to be harmful to humans, were detected (Table 1). Additionally, it should be noted that a significant reduction in the abundance of these potential pathogens in AS was observed, suggesting that the conditions of the activated sludge tank are unfavorable for pathogens. Pathogens that are a risk to public health have been previously identified in wastewater treatment plants. In particular, *Leptospira*, *Mycobacterium* and *Vibrio* have been frequently recovered from waste of wastewater treatment plants [12, 47]. Although enteric pathogens are consistently retrieved from sewage using culture-based approaches, OTUs related to *Escherichia*-*Shigella* were found at very low abundance (0.15% from RS and 0.001% from AS).

**Fig 3:**
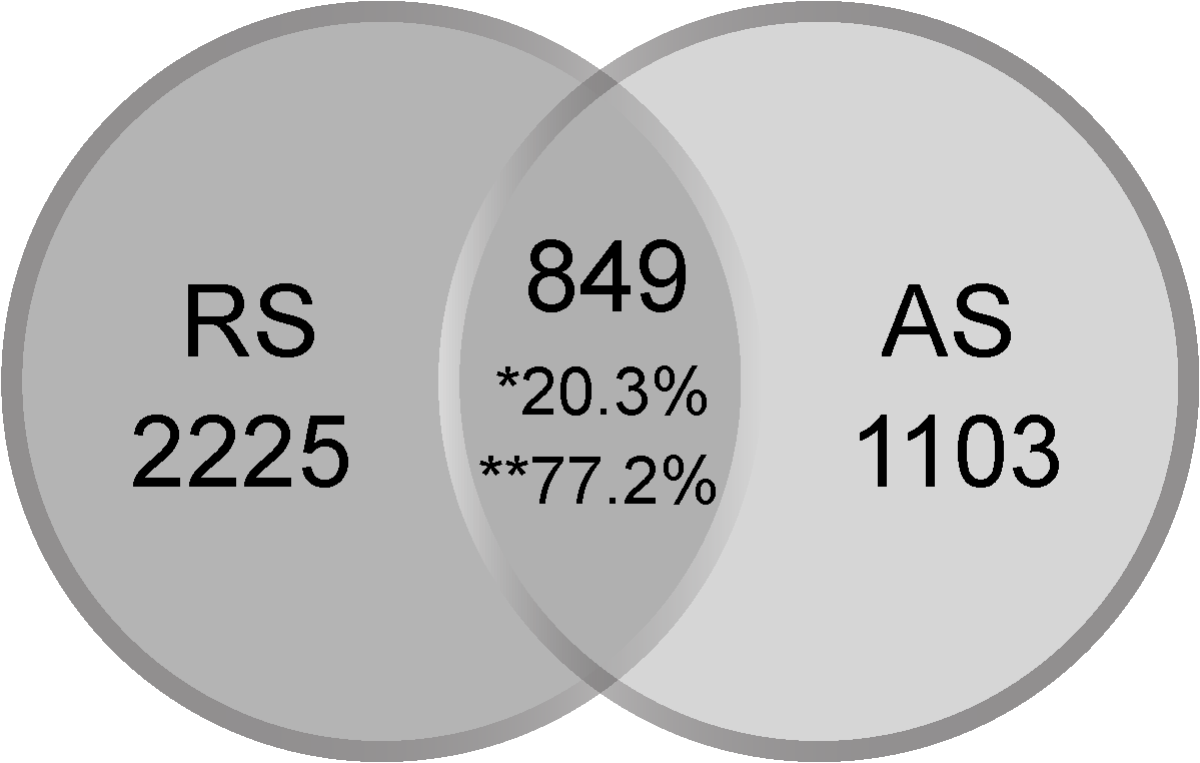
Venn diagram showing the exclusive and shared OTUs from raw sewage (RS) and activated sludge (AS). * % of all OTUs, ** % of all the reads.

The core microbiota was determined using a Venn diagram (Fig 3). A total of 849 OTUs were shared by the two communities, resulting in an overlap of 20.3% of all OTUs. Common OTUs with more than 100 reads were assigned to the Proteobacteria (37 OTUs), Firmicutes (5 OTUs), Bacteroidetes (2 OTUs), Actinobacteria, and Fusobacteria (one OTU each) phyla, comprising 75.6% (RS) and 26.1% (AS) of the reads of these OTUs. Among the core community, the Comamonadaceae, Neisseriaceae, Rhodocyclaceae, Moraxellaceae, Xanthomonadaceae and Aeromonadaceae families were shared by the samples. Moreover, the *Acinetobacter*, *Neisseria*, *Enhydrobacter*, *Dechloromonas*, *Tolumonas* and *Candidatus Accumulibacter* genera were overrepresented in RS. In contrast, most OTUs were only detected in a particular bacterial community. These unique OTUs were largely dominant (72.3%) in relation to the total OTUs in the RS sample but were less important in relation to the relative abundance (2.5%, only 14,638 reads), which is in contrast to the AS sample (43.1%, 253,406 reads). Thus, changes in the community composition between these samples originated from rare OTUs, whereas the core microbiota included strikingly more abundant OTUs.

### Quantitative analysis of the bacterial communities and the *intl1* gene

Molecular tools such as quantitative PCR can effectively measure the amount of bacteria and specific genes, including non-dominant bacteria in various environmental samples [48]. In contrast to studies on environmental samples, many studies in clinical settings have investigated the abundance of class 1 integrons using metagenomic approaches. Sewage and wastewater treatment plants are considered significant sources of resistance genes and mobile elements [49–51]; therefore, these environments are hotspots for the environmental spread of antibiotic resistance mediated by class 1 integrons. Here, the 16S rRNA and *intI1* gene copy numbers of each sample were quantified using qPCR to estimate the proportion of bacteria harboring class 1 integrons.

The bacterial load in the AS sample was higher (2.64 x 10^8^ copies/L) than that in the RS sample (1.23 x 10^8^ copies/L). Moreover, the *intI1* gene copies number per L was 6.54 x 10^7^ in RS and 6.3 x 10^7^ in AS. Thus, the proportion of bacterial cells containing a class 1 integron differed between the samples (RS, 53%; AS, 24%); similar to the results of other studies [50, 52]. Consistent with previous findings [52], we also observed the high removal of bacteria carrying class 1 integrons (55%) after the activated sludge process, suggesting that this process is effective in removing bacteria containing class 1 integrons. Interestingly, an increase in the abundance of the *intI1* gene during the wastewater treatment process has been reported in some studies [53, 54]. The variation of results observed among studies may be attributed to selected bacterial taxa, the climatic and population conditions, occurrence of rain events before sampling as well as organic loading, pH, temperature, dissolved oxygen and sludge retention time applied in the aeration tank. This high removal was accompanied by a decrease in the relative abundance (61%) of Gammaproteobacteria in AS, which could be a consequence of this group’s association with the *intI1* gene. Indeed, it has been suggested that class 1 integrons are broadly distributed throughout Proteobacteria [55].

Our study provides comprehensive insight into the complex bacterial community composition that is associated with sewage treatment plant influent and activated sludge. V6 tag sequencing uncovered not only the dominant taxa but many rare members. Despite the broad taxonomic diversity, only 22.8% of the detected reads were required to explain the differences in community structure between RS and AS. The RS and AS communities were dominated by several taxa that accounted for a large number of reads, and the groups that were found were consistent with those found in all wastewater treatment plant and sewage studies. However, the profiles of the potential pathogens greatly differed among these studies. Our results suggest that class 1 integrons are important outside of the context of Enterobacteriaceae.

## Supporting Information

S1 Fig Standard curves for the 16S rRNA (A) and *intI1* (B) genes. (TIF)

S2 Fig The Ct values of the 16S rRNA (A) and *intI1* (B) *g*enes. (TIF)

S1 Table Summary of the dataset quality control from the raw sewage (RS) and activated sludge (AS) libraries. (DOCX)

S2 Table General features of the raw sewage (RS) and activated sludge (AS) libraries. (DOCX)

S3 Table Taxonomic affiliation of 16S rRNA gene OTUs based on Silva database. (XLSX)

## References

[1] Serviço de saneamento básico é precário em todo o Brasil. Jornal VC/ on line–Brasil. 2012. Available: http://www.tratabrasil.org.br/content/16/08/2012.

[2] Naidoo S, Olaniran AO. Treated wastewater effluent as a source of microbial pollution of surface water resources. Int J Environ Res Publ Health. 2014; 11: 249–270. 10.3390/ijerph110100249 PMC3924443 24366046

[3] Hawkey PM, Jones AM. The changing epidemiology of resistance. J Antimicrob Chemother. 2009; 64: 3–10. 10.1093/jac/dkp256 19675017

[4] Gillings MR, Gaze WH, Prudent A, Smalla K, Tiedje JM, Zhu Y-G. Using the class 1 integron-integrase gene as a proxy for anthropogenic pollution. ISME J. 2014; 1–11. 10.1038/ismej.2014.226 PMC4438328 25500508

[5] Pruden A, Joakim Larsson DG, Amezquita A, Collignon P, Brandt KK, Graham DW, et al Management options for reducing the release of antibiotics and antibiotic resistance genes to the environment. Environ Health Persp. 2013; 121: 878–885. 10.1289/ehp.1206446 PMC3734499 23735422

[6] Poeta P, Radhouani H, Igrejas G, Gonçalves A, Carvalho C, Vinué L, et al Seagulls of the Berlengas natural reserve of Portugal as carriers of fecal *Escherichia coli* harboring CTX-M and TEM extended-spectrum ß-lactamases. Appl Environ Microbiol. 2008; 74: 7439–7441. 10.1128/AEM.00949-08 18835997 PMC2592940

[7] Power ML, Emery S, Gillings MR. Into the wild: dissemination of antibiotic resistance determinants via a species recovey program. PlosOne. 2013; 8(5): e63017. 10.1371/journal.pone.0063017 PMC3661720 23717399

[8] Hu M, Wang X, Wen X, Xia Y. Microbial community structures in different wastewater treatment plants as revealed by 454-pyrosequencing analysis. Bioresour Technol. 2012; 117: 72–79. 10.1016/j.biortech.2012.04.061 22609716

[9] Von Sperling M. Princípios do tratamento biológico de águas residuária**s** In Princípios Básicos do Tratamento de Esgotos, 1st ed., Editora UFMG, Departamento de Engenharia Sanitária e Ambiental–UFMG, Belo Horizonte, Brasil; 1996.

[10] Ferreira FD, Coraiola M. Eficiência do lodo ativado em fluxo contínuo para tratamento de esgoto. Revista Acadêmica Ciência Agrária e Ambiental. 2008; 6: 259–279.

[11] Zhang T, Shao MF, Ye L. 454 Pyrosequencing reveals bacterial diversity of activated sludge from 14 sewage treatment plants. ISME J. 2012; 6: 1137–1147. 10.1038/ismej.2011.188 22170428 PMC3358032

[12] Ye L, Zhang T. Bacterial communities in different sections of a municipal wastewater treatment plant revealed by 16S rDNA 454 pyrosequencing. Appl Microbiol Biotechnol. 2013; 97: 2681–2690. 10.1007/s00253-012-4082-4 22555912 PMC3586070

[13] Cardinali-rezende J, Araújo JC, Almeida PGS, Chernicharo CAL, Sanz JL, Nascimento AMA. Organic loading rate and food-to-microorganism ratio shape prokaryotic diversity in a demo-scale up-flow anaerobic sludge blanket reactor treating domestic wastewater. *A*. Van Leeuw J Microb. 2013; 104: 993–1003. 10.1007/s10482-013-0018-y 24000090

[14] Gillings M, Boucher Y, Labbate M, Holmes A, Krishnan S, Holley M, et al The evolution of class 1 integrons and the rise of antibiotic resistance. J Bacteriol. 2008; 190: 5095–5100. 10.1128/JB.00152-08 18487337 PMC2447024

[15] McLellan SL, Huse SM, Mueller-Spitz SR, Andreishcheva EN, Sogin ML. Diversity and population structure of sewage-derived microorganisms in wastewater treatment plant influent. Environ Microbiol. 2010; 12: 378–392. 10.1111/j.1462-2920.2009.02075.x 19840106 PMC2868101

[16] Zhou H-W, Li D-F, Tam NF-Y, Jiang X-T, Zhang H, Sheng H-F, et al BIPES, a cost-effective high-throughput method for assessing microbial diversity. ISME J. 2011; 5: 741–749. 10.1038/ismej.2010.160 20962877 PMC3105743

[17] Schloss PD, Westcott SL, Ryabin T, Hall JR, Hartmann M, Hollister EB, et al Introducing mothur: open-source, platform-independent, community-supported software for describing and comparing microbial communities. Appl Environ Microbiol. 2009; 75: 7537–7541. 10.1128/AEM.01541-09 19801464 PMC2786419

[18] Quast C, Pruesse E, Yilmaz P. The SILVA ribosomal RNA gene database project: improved data processing and web-based tools. Nucleid Acids Res. 2013; 41: 590–596. 10.1093/nar/gks1219 PMC3531112 23193283

[19] Edgar RC, Haas BJ, Clemente JC, Quince C, Knight R. UCHIME improves sensitivity and speed of chimera detection. Bioinformatics. 2011; 27: 2194–2200. 10.1093/bioinformatics/btr381 21700674 PMC3150044

[20] McMurdie PJ, Holmes S. Basic storage, access, and manipulation of phylogenetic sequencing data with phyloseq. 2012. Available: https://github.com/joey711/phyloseq.

[21] R Core Team. R: A language and environment for statistical computing, 2012. R Foundation for Statistical Computing, Vienna, Austria. ISBN 3-900051-07-0, Available: http://www.R-project.org/.

[22] Raskin L, Stromley JM, Rittmmann BE, Stahl DA. Group- specific 16S rRNA hybridization probes to describe natural communities of methanogens. Appl Environ Microbiol. 1994; 60: 1232–1240. 7517128 10.1128/aem.60.4.1232-1240.1994 PMC201464

[23] Muyzer G, De Waal EC, Uitterlinden AG. Profling of complex microbial population by denaturing gradient gel electrophoresis analysis of polymerase chain reaction-amplified genes coding for 16S. Appl Environ Microbiol.1993; 59: 695–700. 7683183 10.1128/aem.59.3.695-700.1993 PMC202176

[24] Rosewarne CP, Pettigrove V, Stokes HW. Class 1 integrons in benthic bacterial communities: abundance, association with Tn402-like transposition modules and evidence for co-selection with heavy-metal resistance. FEMS Microbiol Ecol. 2010; 72: 35–46. 10.1111/j.1574-6941.2009.00823.x 20132306

[25] Reis MP, Barbosa FA, Chartone-Souza E, Nascimento AMA. The prokaryotic community of a historically mining-impacted tropical stream sediment is as diverse as that from a pristine stream sediment. Extremophiles. 2013; 17: 301–309. 10.1007/s00792-013-0517-9 23389654

[26] Staroscik A. Calculator for determining the number of copies of a template. 2004. Available: http://www.uri.edu/research/gsc/resources/cndna.html.

[27] Hardwick SA, Stoke HW, Findlay S, Taylor M, Gillings MR. Quantification of class 1 integron abundance in natural environments usinf real-time quantitative PCR. FEMS Microbiol Lett. 2008; 278: 207–212. 18042230 10.1111/j.1574-6968.2007.00992.x

[28] Ye L, Shao MF, Zhang T, Tong AH, Lok S. Analysis of the bacterial community in a laboratory-scale nitrification reactor and a wastewater treatment plant by 454-pyrosequencing. Water Res. 2011; 45: 4390–4398. 10.1016/j.watres.2011.05.028 21705039

[29] Metcalf E. Wastewater engineering treatment, disposal and reuse. 3rd ed., Mc Graw Hill, Nova York, USA, 1991.

[30] Yadav TC, Khardenavis A, Kapley AK. Shifts in microbial community in response to dissolved oxygen levels in activated sludge. Bioresource Technol. 2014; 165: 257–264. 10.1016/j.biortech.2014.03.007 24684815

[31] Manz W, Wagner M, Amann R, Schleifer KH. In situ characterization of the microbial consortia active in two wastewater treatment plants. Water Res. 1994; 28: 1715–1723.

[32] Snaidr J, Amann R, Huber I, Ludwig W, Schleifer KH. Phylogenetic analysis and in situ identification of bacteria in activated sludge. App Environ Microb. 1997; 63: 2884–2896. 10.1128/aem.63.7.2884-2896.1997 PMC168584 9212435

[33] Wagner M, Loy A. Bacterial community composition and function in sewage treatment systems. Curr Opin Biotech. 2002; 13: 218–227. 12180096 10.1016/s0958-1669(02)00315-4

[34] Tabrez Khan S, Hiraish A. *Diaphorobacter nitroreducens* gen.nov. sp, nov. a poly(3-hydroxybutyrate)-degrading denitrifying bacterium isolated from activated sludge. J Gen Appl Microbiol. 2002; 48: 299–308. 12682868 10.2323/jgam.48.299

[35] Staley JT, Irgens RL, Brenner DJ *Enhydrobacter aerosaccus* gen. nov., sp. nov. a gas-vacuolated, facultatively anaerobic, heterotrophic rod. Int J Syst Bacteriol. 1987; 37: 289–291.

[36] Shanks OC, Newton RJ, Kelty CA, Huse SM, Sogin ML, McLellan SL. Comparison of the microbial community structures of untreated wastewaters from different geographic locales. App Environ Microb. 2013; 79: 2906–2913. 10.1128/AEM.03448-12 PMC3623150 23435885

[37] Corpe WA, Jensen TE. The diversity of bacteria, eukaryotic cells and viroses in an oligotrophic lake. Appl Microbiol Biot. 1996; 46: 622–630.

[38] Pang CM, Liu W-T. Biological filtration limits carbon availability and affects downstream biofilm formation and community structure. App Environ Microb. 2006; 72: 5702–5712. 10.1128/AEM.02982-05 PMC1563633 16957184

[39] Shimkets LJ, Dworkin M, Reichenbach H. The myxobacteria In: The Prokaryotes: a Handbook on the Biology of Bacteria, 3rd ed., Dworkin M., Falkow S., Rosenberg E., Schleifer K. H. & Stackebrandt E. New York, USA, Springer 2006; 7: 31–115.

[40] Osaka T, Ebie Y, Tsuneda S, Inamori Y. Identification of the bacterial community involved in methane-dependent denitrification in activated sludge using DNA stable-isotope probing. FEMS Microbiol Ecol. 2008; 64: 494–506. 10.1111/j.1574-6941.2008.00473.x 18459970

[41] Wilen BM, Onuki M, Hermansson M, Lumley D, Mino T. Microbial community structure in activated sludge floc analysed by fluorescence in situ hybridization and its relation to floc stability. Water Res. 2008; 42: 2300–2308. 10.1016/j.watres.2007.12.013 18206208

[42] Kirchman DL. The ecology of *Cytophaga–Flavobacteria* in aquatic environments. FEMS Microbiol Ecol. 2002; 39: 91–100. 10.1111/j.1574-6941.2002.tb00910.x 19709188

[43] Nielsen PH, Saunders AM, Hansen AA, Larsen P, Nielsen JL. Microbial communities involved in enhanced biological phosphorus removal from wastewater–a model system in environmental biotechnology. Curr Opin Biotech. 2012; 23: 452–459. 10.1016/j.copbio.2011.11.027 22197171

[44] Mehta-Kolte MG, Bond DR. *Geothrix fermentans* secretes two different redox-active compounds to utilize electrom acceptors across a wide range of redox potentials. App Environ Microb. 2012; 78: 6987–6995. 10.1128/AEM.01460-12 PMC3457516 22843516

[45] Shimizu Y. Microalgal metabolites: a new perspective. Annu Rev Microbiol. 1996; 50: 431–465. 8905087 10.1146/annurev.micro.50.1.431

[46] Molina Grima E, Belarbi E-H, Acien Fernandez FG. Recovery of microalgal biomass and metabolites: process options and economics. Biotechnol Adv. 2003; 20: 491–515. 14550018 10.1016/s0734-9750(02)00050-2

[47] Cai L, Zhang T. Detecting human bacterial pathogens in wastewater treatment plants by a high-throughput shotgun sequencing technique. Environ Sci Technol. 2013; 47: 5433–5441. 10.1021/es400275r 23594284

[48] Wéry N, Lhoutellier C, Ducray F, Delgenés JP, Godon JJ. Behaviour of pathogenic and indicator bacteria during urban wastewater treatment and sludge composting, as revealed by quantitative PCR. Water Res. 2008; 42: 53–62. 17659319 10.1016/j.watres.2007.06.048

[49] Tennstedt T, Szczepanowski R, Braun S, Pühler A, Schlüter A. Occurrence of integron-associated resistance gene cassettes located on antibiotic resistance plasmids isolated from a wastewater treatment plant. FEMS Microbiol Ecol. 2003; 45: 239–252. 10.1016/S0168-6496(03)00164-8 19719593

[50] Zhang X-X, Zhang T, Zhang M, Fang HHP, Cheng S-P. Characterization and quantification of class 1 integrons and associated gene cassettes in sewage treatment plants. Appl Microbiol Biotechnol. 2009; 82: 1169–1177. 10.1007/s00253-009-1886-y 19224208

[51] Pellegrini C, Celenza G, Segatore B, Bellio P, Setacci D, Amicosante G, et al Occurrence of class 1 and 2 integrons in resistant enterobacteriaceae collected from a urban wastewater treatment plant: first report from central Italy. Microb Drug Resist. 2011; 17: 229 *–* 234. 10.1089/mdr.2010.0117 21268680

[52] Ma L, Zhang X-X, Zhao F, Wu B, Cheng S, Yang L. Sewage treatment plant serves as a hot-spot reservoir of integrons and gene cassettes. J Environ Biol. 2013; 34: 391–399. 24620610

[53] Stalder T, Barraud O, Jove T, Casellas M, Gaschet M, Dagot C, et al Quantitative and qualitative impact of hospital effluent on dissemination of the integron pool. ISME J. 2014; 8: 768–777. 10.1038/ismej.2013.189 24152716 PMC3960533

[54] LaPara TM, Burch TR, McNamara PJ, Tan DT, Yan M, Eichmillers JJ. Tertiary-Treated Municipal Wastewater is a Significant Point Source of Antibiotic Resistance Genes into Duluth-Superior Harbor. Environ Sci Technol. 2011, 45: 9543–9549. 10.1021/es202775r 21981654

[55] Stokes HW, Gillings MR. Gene flow, mobile genetic elements and the recruitment of antibiotic resistance genes into Gram negative pathogens. FEMS Microbiol Rev. 2011; 35: 790–819. 10.1111/j.1574-6976.2011.00273.x 21517914

