# Supplementary figures and images for: The Microbiota and Abundance of the Class 1 Integron-Integrase Gene in Tropical Sewage Treatment Plant Influent and Activated Sludge
Source: PLoS One. 2015 Jun 26;10(6):e0131532. doi: 10.1371/journal.pone.0131532 (PMC4482650; doi:10.1371/journal.pone.0131532)

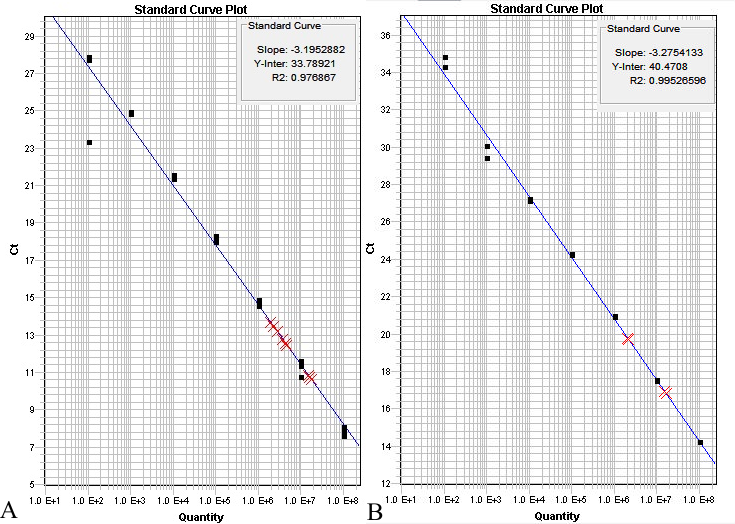

Supplement: S1 Fig — (TIF) [file pone.0131532.s001.tif]

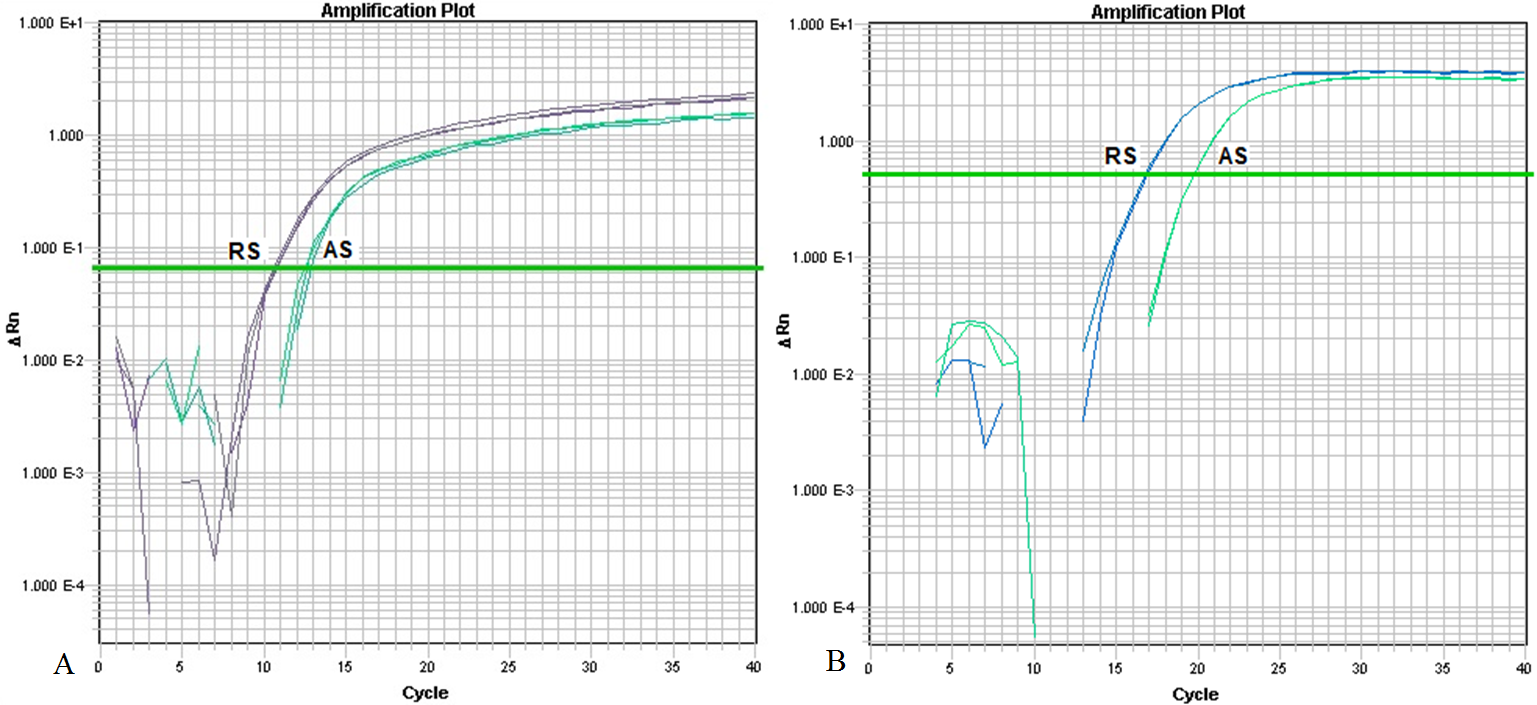

Supplement: S2 Fig — (TIF) [file pone.0131532.s002.tif]
