# Supplementary material for: The Microbiota and Abundance of the Class 1 Integron-Integrase Gene in Tropical Sewage Treatment Plant Influent and Activated Sludge
Source: PLoS One. 2015 Jun 26;10(6):e0131532. doi: 10.1371/journal.pone.0131532 (PMC4482650; doi:10.1371/journal.pone.0131532)
Supplement: S1 Table — (DOCX) [file pone.0131532.s003.docx]

S1 Table. Summary of the dataset quality control from the raw sewage (RS) and activated sludge (AS) libraries.

| Workflow | **RS** | **AS** |
| --- | --- | --- |
| *Raw reads | 943,000 | 942,944 |
| *Resulting reads after a quality cutoff of ≤20 | 609,989 | 616,346 |
| Contigs | 607,164 | 611,547 |
| Resulting reads after removal of homopolymers, ambiguous bases and length outside the range of ≤56 and ≥63bp | 601,585 | 595,642 |
| Resulting reads after aligning | 598,172 | 587,300 |
| Resulting reads after removal of chimera, mitochondria and chloroplast and those outside the Bacteria domain | 598,119 | 587,243 |

*These steps were performed by BGI.
