# Supplementary material for: The Microbiota and Abundance of the Class 1 Integron-Integrase Gene in Tropical Sewage Treatment Plant Influent and Activated Sludge
Source: PLoS One. 2015 Jun 26;10(6):e0131532. doi: 10.1371/journal.pone.0131532 (PMC4482650; doi:10.1371/journal.pone.0131532)
Supplement: S2 Table — (DOCX) [file pone.0131532.s004.docx]

S2 Table. General features of the raw sewage (RS) and activated sludge (AS) libraries.

| **Sample** | **OTUs** | **Chao1** | **ACE** | **Shannon**  **Index** | **Simpson**  **Index** | **Good’s coverage** |
| --- | --- | --- | --- | --- | --- | --- |
| **RS** | 3,074 | 3677.6 | 3843.4 | 3.96 | 0.93 | 99.85% |
| **AS** | 1,952 | 2346.2 | 2441.0 | 3.9 | 0.95 | 99.9% |
